# Quantifying influences of physiographic factors on temperate dryland vegetation, Northwest China

**DOI:** 10.1038/srep40092

**Published:** 2017-01-09

**Authors:** Ziqiang Du, Xiaoyu Zhang, Xiaoming Xu, Hong Zhang, Zhitao Wu, Jing Pang

**Affiliations:** 1 Institute of Loess Plateau, Shanxi University, Taiyuan, Shanxi 030006, China; 2 College of environmental & Resource Science, Shanxi University, Taiyuan, Shanxi 030006, China

## Abstract

Variability in satellite measurements of terrestrial greenness in drylands is widely observed in land surface processes and global change studies. Yet the underlying causes differ and are not fully understood. Here, we used the GeogDetector model, a new spatial statistical approach, to examine the individual and combined influences of physiographic factors on dryland vegetation greenness changes, and to identify the most suitable characteristics of each principal factor for stimulating vegetation growth. Our results indicated that dryland greenness was predominantly affected by precipitation, soil type, vegetation type, and temperature, either separately or in concert. The interaction between pairs of physiographic factors enhanced the influence of any single factor and displayed significantly non-linear influences on vegetation greenness. Our results also implied that vegetation greenness could be promoted by adopting favorable ranges or types of major physiographical factors, thus beneficial for ecological conservation and restoration that aimed at mitigating environmental degradation.

Drylands, including hyper arid, arid, and semi-arid areas, cover more than 40% of the Earth’s terrestrial surface^1^. In China, drylands of ~4.8 million km^2^ occupy approximately half the size of the whole country^2^ and are of substantial environmental and economic importance^3^. Vegetation is a key support to the dryland ecosystems where agricultural and livestock production is the primary economic activity^4^. Dryland vegetation condition is an effective indicator of land degradation^5^, and are therefore of great interest for the assessment of ecological processes in dryland regions^6^.

Variation in terrestrial greenness in drylands has been well documented on various spatiotemporal scales using satellite-based observations of vegetation indices^4^,^7^,^8^. On a regional to global scale, variability can be explained by global or local climatic changes (*e.g.*, temperature, precipitation, drought, solar radiation)^4^,^9^ and anthropogenic influences (*e.g.,* grazing, irrigation, use of fertilisers, human populations, and economic production)^4^,^10^. Other factors (*e.g.*, fire, CO_2_ fertilisation, and nitrogen deposition) have also played a vital role locally^4^,^11^,^12^. These studies have suggested that multiple mechanisms trigger surface greenness patterns. However, the influence of any single factor and their interactive effects on vegetation change are still actively debated and so far unresolved^8^,^12^. Supporting evidence has been found, indicating that each of the primary drivers can result in different dryland vegetation responses^13^. For instance, Nemani *et al*.^11^ found that water availability, temperature, and radiation limits vegetation growth over 40%, 33%, and 27% of the Earth’s vegetated surface, respectively. Fensholt *et al*.^4^ estimated that greenness trends of global semi-arid areas are controlled primarily (about 50%) by precipitation, 7% by air temperature, and nearly 1% by shortwave radiation. De Jong *et al*.^14^ attributed over 50% of the spatial variance in global productivity to changes in climatology. Piao *et al*.^12^ identified that rising atmospheric CO_2_ concentration and nitrogen deposition explain 85% and 41% of the average growing-season leaf area index (LAI) trend in China. Although these studies identified the relative importance of various drivers, they had not considered the interactive impacts of multiple drivers on vegetation greenness.

Over the years, several approaches have been widely used to investigate the drivers of dryland vegetation changes from time series of Earth observational data. For example, rain-use efficiency (defined as the ratio of aboveground net primary productivity to annual precipitation) has been used to discriminate the influences of non-rainfall-related variations on biological productivity (*e.g.,* anthropogenic influences)^15^. Besides linear regression analysis^10^, another approach also based on linearity between rainfall and productivity, known as residual trends analysis, has been employed to assess the human influences on vegetation changes^16^. Recently, an optimal fingerprint method has been utilised to detect the relative contribution of each external driving factor to the observed changes in vegetation activity^8^. However, a critical limitation of these approaches is their assumption of a strong per-pixel linear relationship between drivers and vegetation productivity for the whole time-series^6^. Actually, rigid statistical criteria of linearity may not exist in complex responses of vegetation growth to climate variables^17^. Consequently, the linear proportionality between precipitation and productivity has been questioned by several studies^18^,^19^. A GeogDetector model (GDM) designed by Wang *et al*.^20^, can use spatial variance to quantify the relative importance of single factors and their implicit interactions with response variables.

The objectives of this study are to (1) identify the main drivers and their relative roles in dryland vegetation changes, (2) discern whether the drivers are independent or dependent on one another for annual greenness variation, and (3) discriminate the most suitable physiographic factor characteristics for vegetation growth, as indicated by the maximum NDVI values.

## Results

### Relative influences of physiographic factors on vegetation

The pure effect of each independent factor on vegetation was extracted by calculating their single *PD* value (the power of determinant, Table 1), which indicated the relative importance of physiographic factors. The higher the *PD* value, the stronger the contribution of the factor to surface greenness changes.

Among different physiographic factors, mean annual precipitation, soil type, and vegetation type presented the highest explanatory powers (all above 30%), followed by mean annual temperature, mean annual sunshine duration, and elevation, with influences of more than 15%. Although mean annual wind velocity, slope degree, and slope aspect all influence the spatial and temporal distribution of the water-thermal conditions, the *PD* value of each single factor on greenness was below 10%, indicating wind, slope, and aspect had only a minimal contribution to the observed greenness changes unless combined with other factors.

### Combined influences of physiographic factors on vegetation

The interactive effect of any two physiographic factors (symbolized by ∩) was analysed by comparing their combined contribution to greenness changes with their individual contributions. The jointly effects were shown by their interactive *PD* values (Table 2). The interactive *PD* values were as follows: MAP∩ST (0.7087) > MAT∩ST (0.6069) > ST∩MASD (0.6043) > VT∩MAT (0.5442) > ST∩MAWV(0.5315) > VT∩MASD (0.5238) > MAP∩slope (0.5177) > MAP∩VT (0.4782). This result showed that the superposition between meteorological factors and micro-topographic conditions greatly enhanced their individual effects on surface greenness. The same phenomenon also existed for the combined effect of wind velocity and slope degree, although it had little effect on the change in greenness (*e.g.,* MAWV∩SD = 0.1546). Similarly, the combined effects of either two climatic factors (*e.g.,* MAP∩MASD = 0.5212, MAT∩MASD = 0.4882, and MAP∩MAT = 0.4867) or two micro-topographic factors (*e.g.,* ST∩VT = 0.6813, ST∩SD = 0.4712, and VT ∩slope = 0.4699) were also included to better understand the contribution to greenness change. It could be clearly seen that all interactive *PD* values of coupled factors were greater than any *PD* value of individual factor. These findings indicated that the effects of physiographic factor on surface greenness were not independent but interact significantly. Furthermore, some interactive effects of physiographic factors manifested a non-linear enhanced relationship (*e.g.,* ST ∩ELE, VT∩SD, MAP∩MAWV, MAT∩MASD, and ST∩MAWV). In addition, there was a strong double-synergy effect of physiographic factors on terrestrial greenness (*e.g.,* MAP∩VT, ST∩VT, ST∩MAT, *et al*.). The findings implied that the interaction of multiple factors was not a simple additive process but a nonlinear interaction that influences vegetation greenness.

### Suitable limits of physiographic factors for vegetation growth

The suitable limits of the main physiographic factors were analysed according to the GDM, and their statistical significance was tested using *t-test* at the 95% confidence level. The characteristics of each physiographic factor with larger NDVI values were assumed to be more suitable for vegetation growth.

Mean annual greenness for different factor showed obviously disparities (Table 3). For instance, the NDVI values increased with increasing precipitation and reached a maximum value when annual precipitation was between 316.20 mm and 504.08 mm, implying that this level of precipitation promoted widespread greenness in the study area. As for elevation, the highest annual mean NDVI value occurred at altitudes between 1727 m and 2896 m, indicating that this was the optimal elevation range for vegetation growth in the study area. However, vegetation growth was impeded (NDVI value below 0.1) when the altitude was above 4272 m, suggesting that this was a threshold elevation. Sunshine duration potentially impacted photosynthesis and vegetation growth. A reduction in NDVI was accompanied by an increase in sunshine duration. Moreover, the highest annual NDVI value of 0.4 roughly corresponded to a sunshine duration between 2293.13 and 2731.42 h/a, indicating that surface greenness was more apparent in this range of sunshine duration. Similar to previous studies^21^, which stressed a crucial role of wind in many plant ecosystems, we found that NDVI values decreased as wind speed increased. In addition, there was an annual NDVI peak under the mean annual wind velocity of 1.81–2.38 m/s. For the mean annual temperature, the highest annual NDVI value occurred between 6.22–8.58 °C. With regard to vegetation type, the highest annual mean NDVI value occurred in coniferous forest, followed by crop vegetation and meadow. Similarly, the highest annual mean NDVI value occurred in chernozems and greyzems, followed by fluvisols, greysols, arenosols, and leptosols, with an annual mean NDVI value above 0.4. These findings demonstrated that vegetation greenness could be promoted by controlling the main influential factors within their corresponding favourable ranges or types.

## Discussion

**Figure 1:**
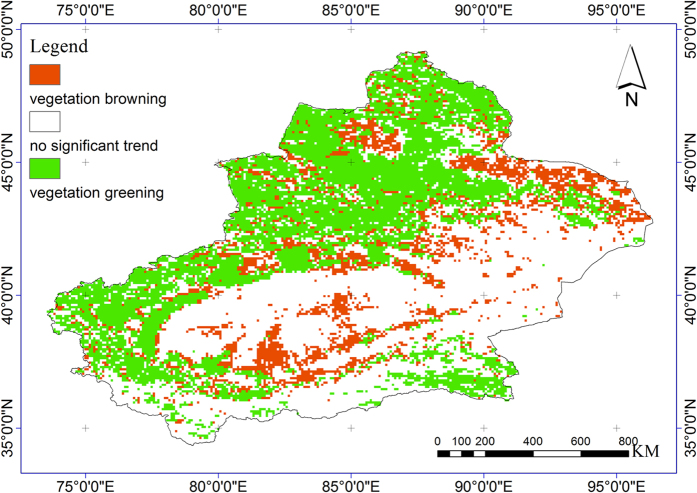
Spatial pattern of vegetation greenness change trend. The map was plotted using ArcGIS 10.2 (http://www.esri.com/).

**Figure 2:**
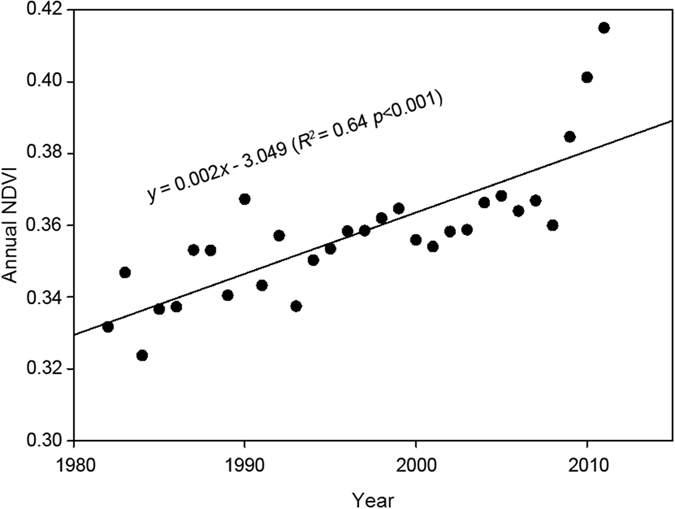
Annual change of vegetation greenness.

Long-term changes in vegetation greenness have been observed since the 1980s. It is generally acknowledged that vegetation greenness has significantly increased both on global^8^ and continental scales^22^, and in worldwide arid areas^4^. In most areas of China, satellite measurements have shown an unequivocal greening trend on average since 1982^23^,^24^,^25^. Similarly, the areas of significant vegetation greening and browning account for approximately 41.27% and 28.12% of the study area (Fig. 1), respectively. Moreover, there has been an increasing trend of 0.002 yr^−1^ (*P* < 0.001) in vegetation cover (Fig. 2). This result agreed with previous conclusions of surface greening in our study area^26^,^27^. Time-series satellite observation-based changes in terrestrial greenness are of great interest for assessment of changes in environmental conditions in dryland ecosystems. However, one challenge remaining in dryland vegetation dynamics researches is disentangling the individual role of potential drivers and their interactions with one another. In our case study, the GDM was conducted to investigate the independent and interactive effects of physiographic factors on the greenness change, as well as the favourable ranges of the main factors influencing vegetation growth. We therefore explicitly assessed the influences of physiographic variables on the greenness of vegetated dryland in Northwest China.

This study indicates that the variation in dryland greenness was predominantly and significantly impacted by precipitation, soil type, vegetation type, and temperature, independently or jointly. However, sunshine duration and elevation showed relatively lower individual influences. Our results also suggested that the variation in vegetation greenness did not be completely explained by wind velocity, slope degree, or slope aspect because these factors had the lowest individual *PD* values. In addition, our work confirmed that physiographic factors functioned together to change surface greenness, and interactions between factors played a more important role than their individual effects on greenness variability. Although the individual effects of slope and wind velocity were weak, these factors made a greater contribution to greenness variability when interacting with precipitation, soil, and temperature.

Although drylands across the globe have experienced an increase in greenness from 1981 to the present, explanations are diverse^6^. Climatic factors are generally regarded as vital biophysical components influencing vegetation growth. Our findings were in agreement with previous studies^27^,^28^, in which annual precipitation was found to exert the strongest local effects on greenness dynamics. It further verified that water availability was the limiting factor in most temperate dryland areas. Compared to precipitation, temperature alone was relatively weak in driving the interannual improvement of greenness. A possible explanation was that increased temperature triggered vegetation growth. Moreover, increased temperature also caused the decline of soil moisture, which adversely affected vegetation growth^29^. Furthermore, we found that the combined effects of MAP and MAT significantly enhanced the effects of temperature on greenness change (*i.e., PD* (MAP ∩ MAT) > *PD* (MAT)). The increased precipitation offseted the enhancement in evapotranspiration resulting from a significantly warming climate. Therefore, the exact coupling between temperature and precipitation was a vital interaction for regulating vegetation growth. This further confirmed that vegetation in temperate drylands was constrained by hydrothermal conditions, unlike in tropical drylands, where vegetation was primarily determined by rainfall^5^.

Solar radiation not only directly affects the photosynthesis of green plants but also indirectly affects surface greenness by means of evapotranspiration and plant available soil moisture. The annual sunshine duration can serve as a reliable proxy for interannual variations in solar radiation^30^. The relatively small *PD* value demonstrated that the effect of sunshine duration on the annual variation in vegetation greenness was very limited, even though there was plentiful sunshine (average 2847.08 hours/a) in our study area. This phenomenon might be attributed to the significant evapotranspiration induced by excessive sunshine, which inhibited vegetation growth.

Wind also appeared to have an indirect impact on vegetation growth conditions by altering local microclimate^21^,^31^. Unlike the aforementioned climate factors, wind velocity showed little influence (*PD* = 0.0897) on increasing greenness in this study. Data from meteorological stations with unbroken records demonstrated that MAWV (about 2.24 m/s) changed little and non-significantly in the last three decades. Such low wind velocity per year would cause an increase in transpiration, resulting in water loss or a general cooling of the temperature, thus an adverse impact on vegetation growth was observed.

Previous studies demonstrated that the effect of soil type on vegetation growth and rain-use efficiency was striking in the areas where rainfall was the limiting factor for primary production^32^,^33^. Comparably, we found that soil type was the second most important factors influencing vegetation greenness, with an individual *PD* value of 0.4261. Annual NDVI values were clearly diverse among different soil types; the largest annual NDVI value was 0.74 in chernozem zones, and the smallest was 0.10 in sandy soil zones. The possible reasoning behind these observed differences in vegetation growth was the different moisture retention characteristics of soil, especially in arid regions where precipitation was the main climatic constraint to vegetation productivity^33^.

The relatively high *PD* value (0.3795) indicated that vegetation type was another important factor. In our study, the different vegetation types displayed different NDVI values. For instance, coniferous forest zones had the highest annual mean NDVI value, while barren deserts showed the lowest annual mean NDVI value. The reason for such discrepancy was that different vegetation types had very different spectral reflectances in the red and near-infrared wavebands, as defined in the NDVI^34^. Another underlying reason was that different vegetation types had different sensitivities to climate factors, and vegetation that was especially sensitive to hydrothermal conditions was likely to fluctuate markedly in NDVI and biomass^35^. For example, Li and Yang^5^ found that desert steppe was most strongly affected by warmer temperatures, hence the decreased NDVI that occurred mainly in drier summers (May and June) in inner Mongolia. However, Zhang *et al*.^36^ found that an increasing summer temperature did not decrease vegetation greenness but instead promoted vegetation growth in our study area.

Compared with physical factors, geographical factors (*e.g*., elevation, slope degree and slope aspect) exerted weak effects on vegetation greenness, and the influence of elevation on vegetation greenness was stronger than that of the slope variables (*e.g*., slope degree and slope aspect). In terms of elevation, we found that the highest annual mean NDVI value at altitudes of 1727~2896 m, while the lowest value occurred at altitudes above 4272 m. This change in vegetation greenness with elevation gradient in the current study might be explained by a large variety of different climates and soils determined by elevation. The altitudinal gradient is correlated to the variation in annual precipitation and mean annual temperature. Water availability is limited at the lower and middle elevations and energy is limited at the higher elevations^37^. Moreover, nutrient limitations often increase with increasing elevation and associated declining temperatures^38^. As a consequence, despite an indirect topographical factor, elevation is considered to influence vegetation patterns through other more direct factors^39^. For example, the *PD* (VT∩ELE) far exceeded both the single *PD* (VT) and *PD* (ELE). The reason for this fact might be that interactive effects between elevation and vegetation type determine the influence of N and P addition on plant and microbial properties^40^.

Similar to elevation, slope degree and slope aspect affect the amount of solar radiation intercepted by the surface and have been identified as the key factors in determining ecological conditions^41^. For example, pronounced discrepancies in soil water content, soil layer depth, the total amount of plant available water, and soil temperature can occur between north- and south-facing slopes^41^,^42^. However, vegetation variation was not well explained by the influence of slope and aspect due to their very small *PD* values (*e.g., PD* (SD) = 0.0699, *PD* (SA) = 0.0099). Although slope and aspect had little influence on vegetation growth, when combined with vegetation type, precipitation, and soil type, they played a vital role in influencing vegetation greenness (*e.g., PD* (MAP∩SD) = 0.5177, *PD* (ST∩SD) = 0.4712, *PD* (VT∩SD) = 0.4699, *PD* (MAP∩SA) = 0.5076, *PD* (ST∩SA) = 0.5192 and *PD* (VT∩SA) = 0.4538). Such interactive *PD* values implied that together they strengthened the independent influences on vegetation greenness.

This study highlighted the advantages of the GDM in detecting the spatial consistency of vegetation greenness with its influential factors. Several approaches, such as optimal fingerprint detection method^8^, rain-use efficiency^15^, residual trends analysis^16^ and classic regression models^10^ were employed to interpret the relationships between greenness and drivers. These models were usually based on some assumptions or restrictions such as normal distribution and linear hypothesis. Compare to previous approaches, the GDM does not have the aforementioned limitations between drivers and response variables^20^,^43^, and can deal with qualitative (*e.g*., soil and vegetation type) and quantitative data (*e.g.*, climatic variables) effectively. Furthermore, it can quantify the complex interactions between explanatory and response variables. This powerful approach can be introduced to other spatial analysis studies.

A better understanding of the influences of physiographic factors on terrestrial greenness is a critical step forward for clarifying the driving mechanisms behind greenness trends. To some extent, our work may be helpful for improving future programs of ecological conservation and restoration in order to mitigate environmental degradation problems. However, there are still some issues to be resolved. For example, in this study, only nine physiographic factors were considered for dryland vegetation, which appears to be insufficient to capture the complexity of greenness change mechanisms. Additional variables should be studied in the future, such as extreme weather events, thawing–freezing processes, atmospheric CO_2_ concentration, nitrogen deposition and their combined effects. Furthermore, human activities (*e.g.,* cultivation, irrigation, and land use change) and socioeconomic forces (*e.g.*, populations, GDP, and income) will also contribute to the ongoing research on surface greenness dynamics.

## Methods

### Study area

This study was undertaken in the Xinjiang Uygur Autonomous Region (75°E to 95°E and 35°N to 50°N, Fig. S1), the largest in the area of all the provincial administrative regions in China. Located on the border of Northwest China, Xinjiang occupies an area of more than 1.66 million km^2^, covering approximately 28% of Central Asia and about 17% of the entire territory of China. Lying in the hinterland of the Eurasian continent, far from the ocean with mountains all around, Xinjiang is one of the driest regions in the world and an important case of a dryland ecosystem in China. The water deficit results from infrequent precipitation and high potential evapotranspiration. The wide variety of physical features and climatic conditions have led to many diverse ecological habitats such as sparse desert vegetation dominated primarily by xeric shrubs (*e.g.*, Haloxylon ammodendron distributed primarily in Junggar basin, and Tamarix ramosissima located mainly in Tarim Basins), alpine meadows and dry-steppe (*e.g*., Stipa baicalensis and Leymus chinensis), natural forest (*e.g*., Populus euphratica located mainly in Tarim Basins), and herbaceous swamp ecosystems. Vegetation productivity is of great economic significance because crop and livestock production is the primary economic activity and it is a key support for the dryland ecosystem since the start of human settlement in the area. Detailed settings of the study area can be found in the previous paper^26^,^27^,^28^,^44^.

### Data description

#### The proxy for greenness dynamics

The long-term normalised difference vegetation index (NDVI), derived from the NOAA Advanced Very High-Resolution Radiometer (AVHRR) satellite record, is currently the most widely used data source for detecting greenness dynamics over large geographical areas^45^. A new generation NDVI data set (NDVI3g) developed by the Global Inventory Monitoring and Modeling Studies group (GIMMS) at NASA’s Goddard Space Flight Center was used as a proxy for surface greenness in this study. The GIMMS NDVI3g data have approximately 8 km spatial resolution and a bi-weekly temporal resolution. It is the most recent and longest lasting global greenness index covering the period from July 1981 to December 2011, and is open to the public via the NASA Ames Ecological Forecasting Lab. The dataset was processed using the geometry, the radiation and the atmospheric correction, and then the daily data and every image were precisely processed by geometric correction, eliminating the cloud and the bad line (for a detailed description refer to Pinzon and Tucker^46^). The original 15-day NDVI3g data were aggregated in ArcGIS10.2 platform to 30-year series using a maximum value composite approach that can further reduce the influences of the atmosphere, clouds, and solar zenith angle^47^. Pixels with an average annual NDVI below 0.1 were assumed to be non-vegetated areas (*e.g*., water and bare surfaces) and thus removed in our analysis^48^.

#### Physiographic factors

Because climate, terrain, soil, and vegetation conditions affect greenness variability^10^,^49^, we considered these factors in our study. Mean annual precipitation (MAP), mean annual temperature (MAT), mean annual sunshine duration (MASD), and mean annual wind velocity (MAWV) were selected as proxy variables for climatic environment. Elevation (ELE), slope degree (SD), and slope aspect (SA) were used as a proxy for the local terrain elements. The proxy parameters for soil and vegetation were soil type (ST) and vegetation type (VT), respectively.

Climatic data from 1982 to 2011 from 57 meteorological stations in Xinjiang Region, containing MAP (Fig. S2), MAT (Fig. S3), MASD (Fig. S4), and MAWV (Fig. S5), were interpolated using the inverse distance weighting method, and were then classified by using the natural breakpoint method^50^. Climatic data were ground station data from the China Meteorological Science Data Sharing Service network.

Topographic factors including the ELE (Fig. S6), SD (Fig. S7), and SA (Fig. S8) were extracted from the 90 m SRTM DEM, provided by the Cold and Arid Regions Sciences Data Center at Lanzhou (http://westdc.westgis.ac.cn).

Soil type data were collected from the China soil map-based harmonised world soil database (v1.1). The data set was provided by the Cold and Arid Regions Sciences Data Center at Lanzhou (http://westdc.westgis.ac.cn) (Fig. S9).

Vegetation type data were available from the vegetation map of China (scale 1:4000000). The data set was provided by the Environmental and Ecological Science Data Center for West China, National Natural Science Foundation of China (http://westdc.westgis.ac.cn) (Fig. S10).

All the continuous data *(e.g.*, MAP, MAT, MAWV, SD, ELE and SA) were discretized into different categorical zones using prior knowledge or the optimal classification methods (*e.g.,* natural breaks, *etc*.) according to their spatial heterogeneity^50^,^51^. Then, all the physiographic factors or their proxy variables from diverse sources were adjusted into uniform pixel size and projection constrained by the same boundary of the study area by creating a fishnet of 1 km × 1 km scale in ArcGIS 10.2 Desktop software^52^. Finally, all data derived from intersecting calculations preprocessed with ArcGIS 10.2 were input into Excel-Geodetector^52^.

#### GeogDetector model (GDM) description

A new spatial analysis method, GDM was originally used to detect the influences of environmental factors on human health^20^,^43^,^53^. The maxim of GDM is that a relevant outcome *Y* would exhibit a similar spatial distribution to its impact factor *X* in geographical space^54^. In our study, we assume that if some physiographic factors affect on the variations in vegetation greenness, then the spatial distribution of these factors and vegetation greenness may show greater consistency.

The degree of spatial correspondence between *Y (e.g.*, vegetation greenness) and *X (e.g.*, physiographic factors) is measured by the power of determinant (*PD*) for a factor, which can be expressed as


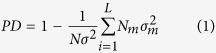


where *N* and *σ*^2^ is the number of total samples and global variance of *Y* over the entire study area, respectively. *N*_*m*_ is number of samples in zone *m*, 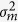 is the variance of *Y* within zone *m* of the factor *X*, and *L* is the number of zones (categories) of the factor *X*. The definition of *σ*^2^ and 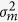 apply here.


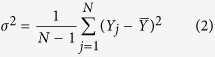


where *Y*_*j*_ and 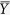
is the value of the *j*th sample and the global mean of *Y* over the entire study area, respectively.


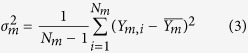


where *Y*_*m,i*_ and 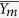 is the value of *i*th sample and the mean of *Y* in zone *m*, respectively.

In equation (1), *PD* ∈ [0,1] means that if the factor *X* does not affect the spatial pattern of *Y, PD* = 0, while if the factor *X* completely controls factor *Y*, then *PD* = 1. Higher values of *PD* indicate higher spatial association between *X* and *Y*.

The GDM can also be applied to probe the interaction between any two factors (symbolized by ∩). If *PD (X*_1_∩*X*_2_) > *PD (X*_1_) or *PD (X*_1_∩*X*_2_) > *PD (X*_2_), the factors enhance each other; if *PD (X*_1_∩*X*_2_) > *PD (X*_1_) and *PD (X*_1_∩*X*_2_) > *PD (X*_2_), and *PD (X*_1_) + *PD (X*_2_) > *PD (X*_1_∩*X*_2_), the factors bienhance each other; if *PD (X*_1_∩*X*_2_) > *PD (X*_1_) + *PD (X*_2_), the factors nonlinearly enhance each other. If the less-than signs “<” is in previous situations, the two factors respectively weaken, biweaken, or nonlinearly weaken each other. If *PD (X*_1_∩*X*_2_) = *PD (X*_1_) + *PD (X*_2_), then they are independent of each other. Thus, the individual and interactive *PD* value can be used to quantify influences of physiographic factors on vegetation greenness.

More details on GDM can be found in the original paper^20^, here, we briefly review the model within our present context. The free software for GDM can be downloaded from http://www.sssampling.org/geogdetector/.

All data were originally stored in an Excel file: the first column stored sample data (*Y*), the following columns stored its impact factors (*X*). Download a version of Excel-GDM, read data and run, a worksheet “Factor detector” was created and its *PD* value was presented^55^.

## Additional Information

**How to cite this article**: Du, Z. *et al*. Quantifying influences of physiographic factors on temperate dryland vegetation, Northwest China. *Sci. Rep.* **7**, 40092; doi: 10.1038/srep40092 (2017).

**Publisher's note:** Springer Nature remains neutral with regard to jurisdictional claims in published maps and institutional affiliations.

## Supplementary Material

Supplementary Figures

## Figures and Tables

**Table 1:** *PD* values of physiographic factors.

Physiographic factors	MAP	ST	VT	MAT	MASD
***PD*** **values**	0.4559	0.4261	0.3795	0.2227	0.1942
**Physiographic factors**	ELE	MAWV	SD	SA	
***PD*** **values**	0.1593	0.0897	0.0699	0.0099	

MAP = mean annual precipitation; ST = soil type; VT = vegetation type; MAT = mean annual temperature; MASD = mean annual sunshine duration; ELE = Elevation; MAWV = mean annual wind velocity; SD = slope degree; SA = slope aspect.

**Table 2:** Interactive *PD* values between pairs of physiographic factors.

C = *PD (x*_*1*_∩*x*_*2*_)	A = *PD (x*_*1*_)	B = *PD (x*_*2*_)	D = *PD (x*_*1*_) + *PD (x*_*2*_)	Conclusion	Interpretation
MAP ∩ VT = 0.7087	0.4559	0.3795	0.8354	C<D; C>A,B	↑
ST ∩ VT = 0.6813	0.4261	0.3795	0.8056	C<D; C>A,B	↑
ST ∩ MAT = 0.6069	0.4261	0.2227	0.6488	C<D; C>A,B	↑
ST ∩ MASD = 0.6043	0.4261	0.1942	0.6203	C<D; C>A,B	↑
ST ∩ ELE = 0.6010	0.4261	0.1593	0.5854	C>D; C>A,B	↑↑
VT ∩ MAT = 0.5442	0.3795	0.2227	0.6022	C<D; C>A,B	↑
VT ∩ ELE = 0.5328	0.3795	0.1593	0.5388	C<D; C>A,B	↑
ST ∩ MAWV = 0.5315	0.4261	0.0897	0.5158	C>D; C>A,B	↑↑
VT ∩ MASD = 0.5238	0.3795	0.1942	0.5737	C<D; C>A,B	↑
MAP ∩ MASD = 0.5212	0.4559	0.1942	0.6501	C<D; C>A,B	↑
MAP ∩ SD = 0.5177	0.4559	0.0699	0.5258	C<D; C>A,B	↑
MAT ∩ MASD = 0.4882	0.2227	0.1942	0.4169	C>D; C>A,B	↑↑
MAP ∩ MAT = 0.4867	0.4559	0.2227	0.6786	C<D; C>A,B	↑
MAP ∩ VT = 0.4782	0.4559	0.3795	0.8354	C<D; C>A,B	↑
MAP ∩ MAWV = 0.4763	0.4559	0.0897	0.5456	C>D; C>A,B	↑↑
ST ∩ SD = 0.4712	0.4216	0.0699	0.4915	C<D; C>A,B	↑
VT ∩ SD = 0.4699	0.3795	0.0699	0.4494	C>D; C>A,B	↑↑

“↑” denotes *x_1_* and *x_2_* enhance each other; “ ↑↑” denotes a non-linear enhancement of *x_1_* and *x_2_*. The physiographic factors include: mean annual precipitation (MAP), vegetation type (VT), soil type (ST), mean annual temperature (MAT), mean annual sunshine duration (MASD), mean annual wind velocity (MAWV), slope degree (SD) and elevation (ELE).

**Table 3:** Suitable limits of main physiographic factors (95% confidence level).

Physiographic factors	Suitable limits	Mean annual NDVI
Mean annual precipitation (mm)	316.21~504.08	0.67
Soil type	Chernozems	0.74
	Gypsisols	0.68
Vegetation type	Coniferous forest	0.64
	Crop vegetation	0.51
	Meadow	0.43
Mean annual temperature (°C)	0~5	0.43
Mean annual Sunshine duration (h/a)	2293.12~2732.42	0.41
Elevation* (m)	1727~2896	0.41
