# Supplementary material for: Quantifying influences of physiographic factors on temperate dryland vegetation, Northwest China
Source: Sci Rep. 2017 Jan 9;7:40092. doi: 10.1038/srep40092 (PMC5220299; doi:10.1038/srep40092)
Supplement: Supplementary Figures [file srep40092-s1.pdf]

Quantifying influences of physiographic factors on temperate dryland vegetation, Northwest China

Ziqiang Du[1,*], Xiaoyu Zhang[2], Xiaoming Xu[1], Hong zhang[2], Zhitao Wu[1], Jing Pang[2]

[1]Institute of Loess Plateau, Shanxi University, Taiyuan, Shanxi 030006, China; [2]College of environmental ＆Resource Science, Shanxi University, Taiyuan, Shanxi 030006, China. [*] Correspondence and requests for materials should be addressed to Z. D. (duzq@sxu.edu.cn)

Supplementary information

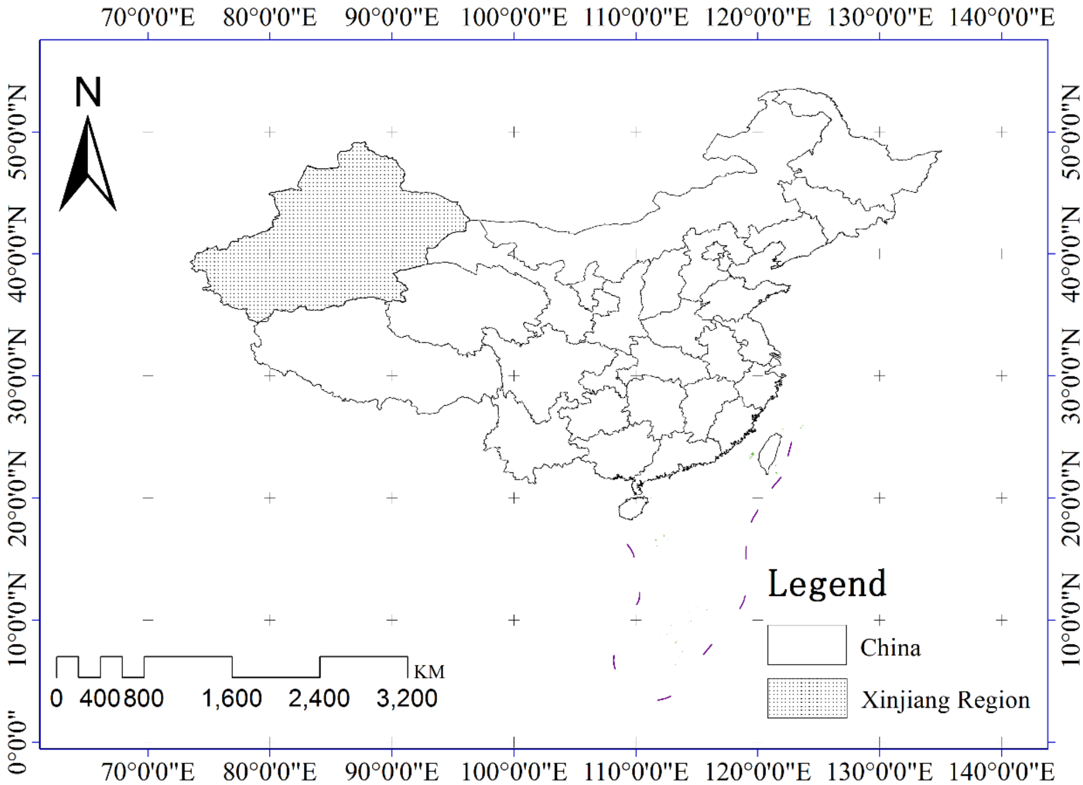

Fig. S1 Location of the study area. The map was plotted using ArcGIS 10.2 (http://www.esri.com/).

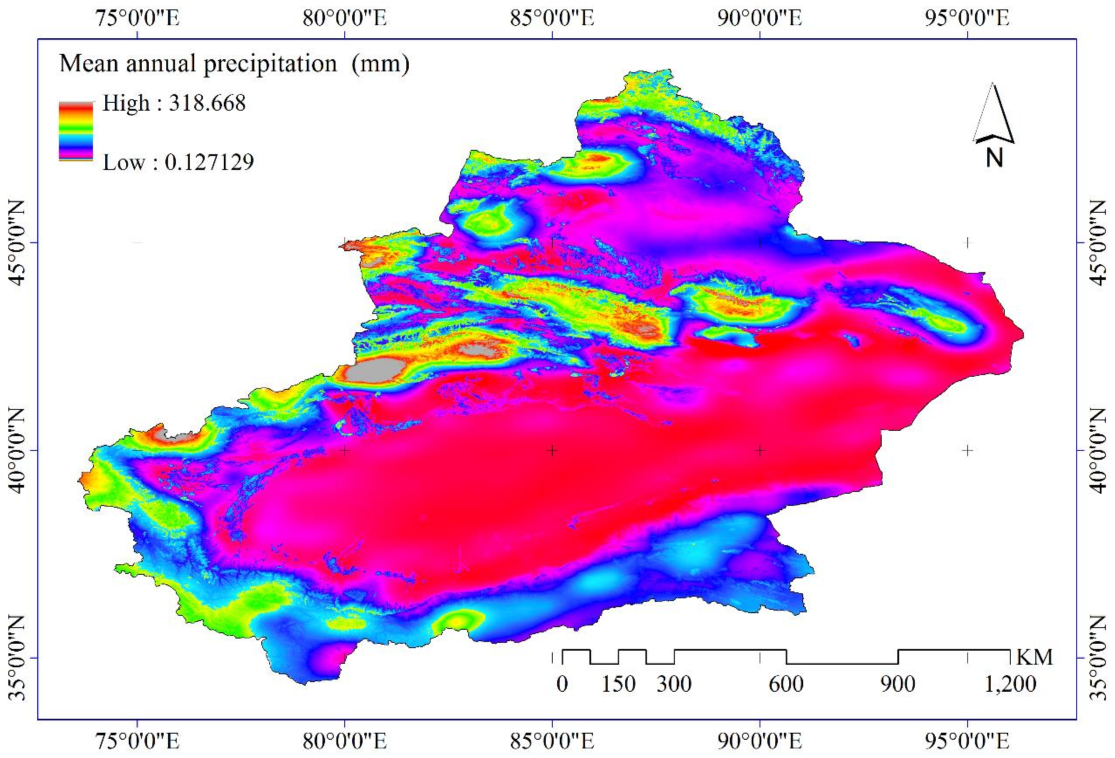

Fig. S2 Mean annual precipitation. The map was plotted using ArcGIS 10.2 (http://www.esri.com/).

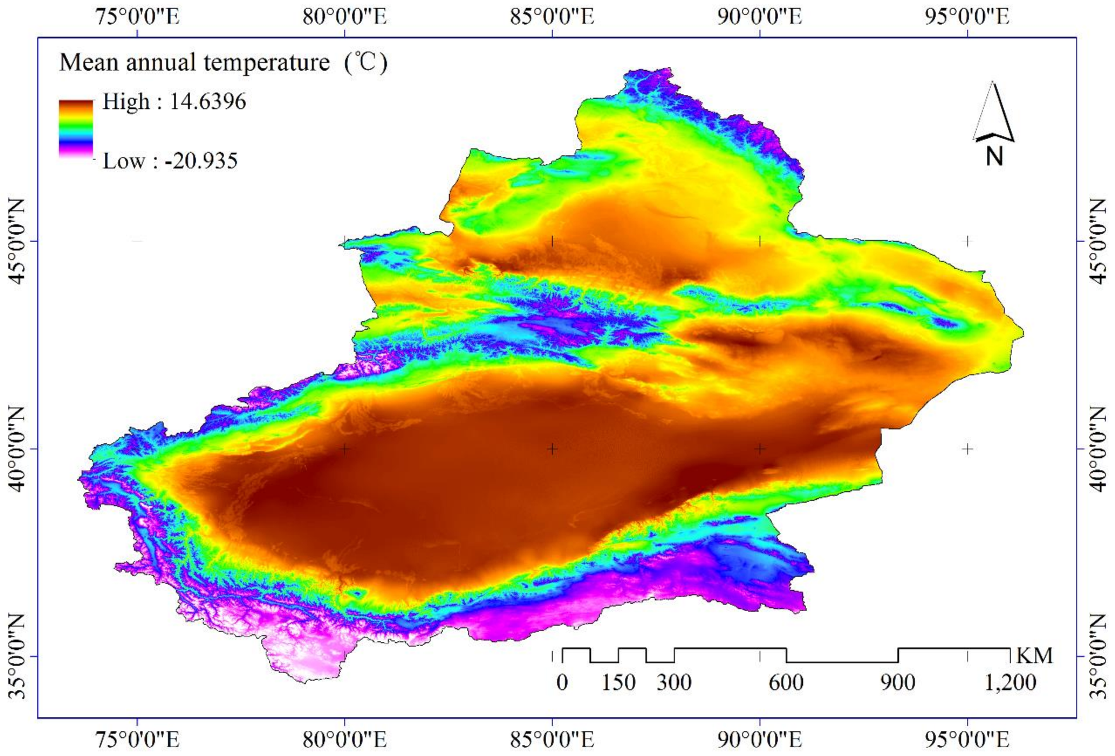

Fig. S3 Mean annual temperature. The map was plotted using ArcGIS 10.2 (http://www.esri.com/).

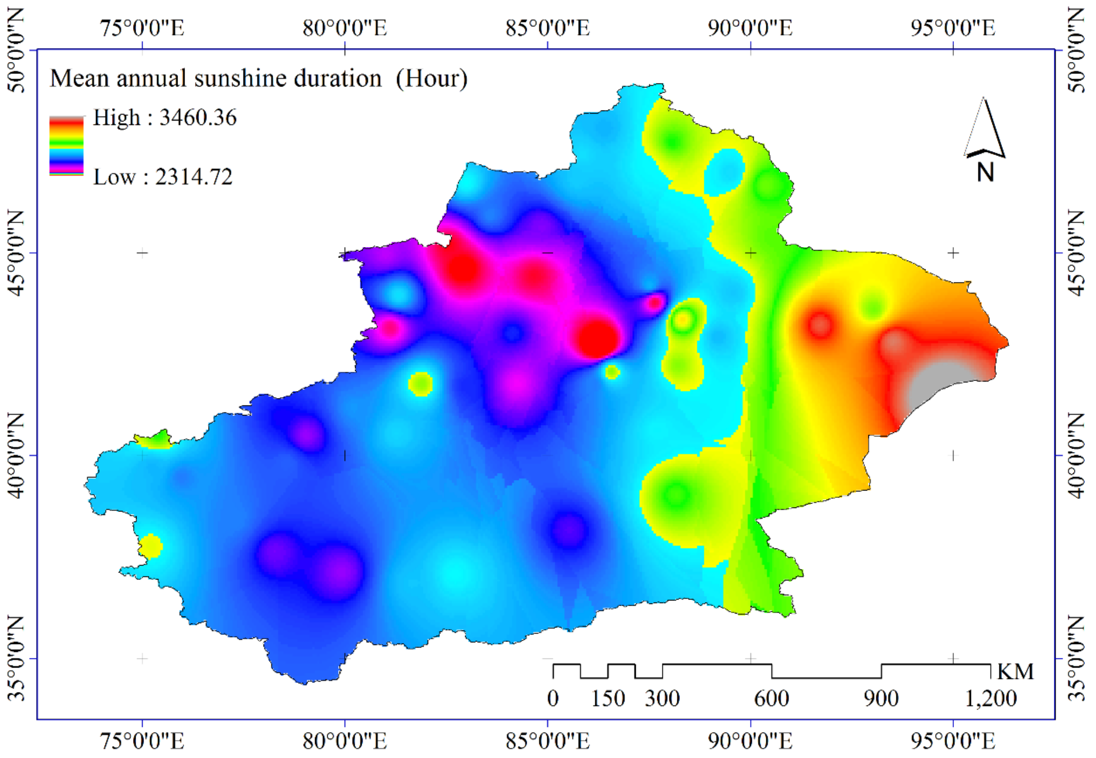

Fig. S4 Mean annual sunshine duration. The map was plotted using ArcGIS 10.2 (http://www.esri.com/).

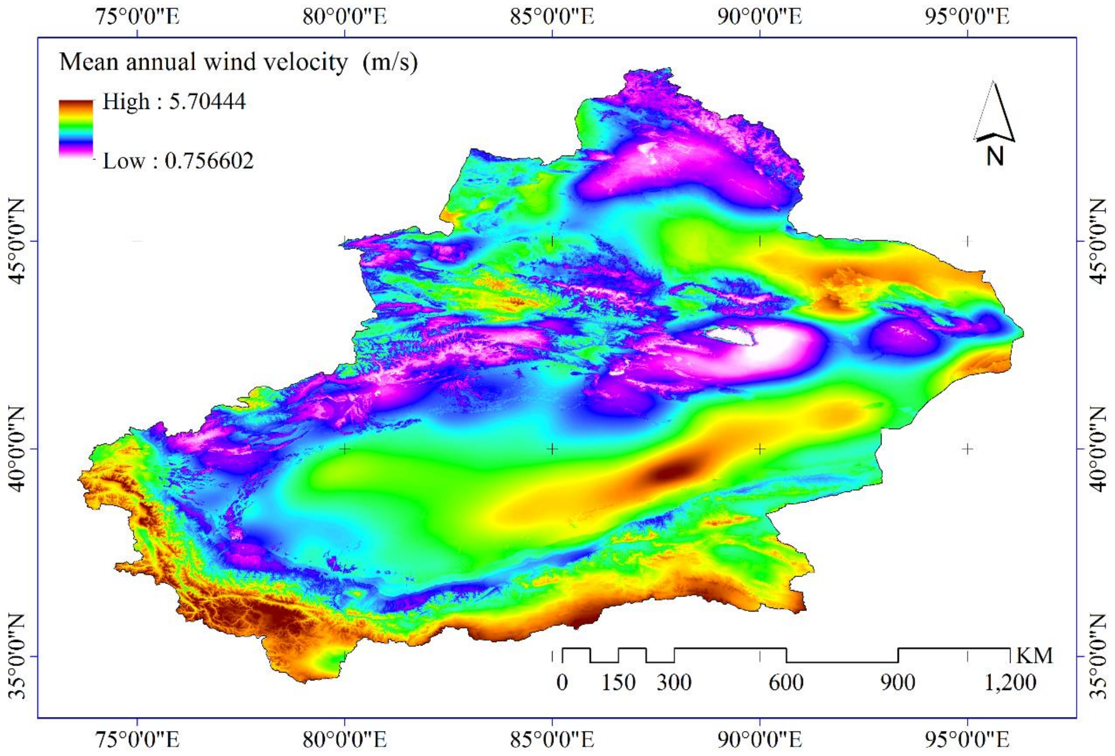

Fig. S5 Mean annual wind velocity. The map was plotted using ArcGIS 10.2 (http://www.esri.com/).

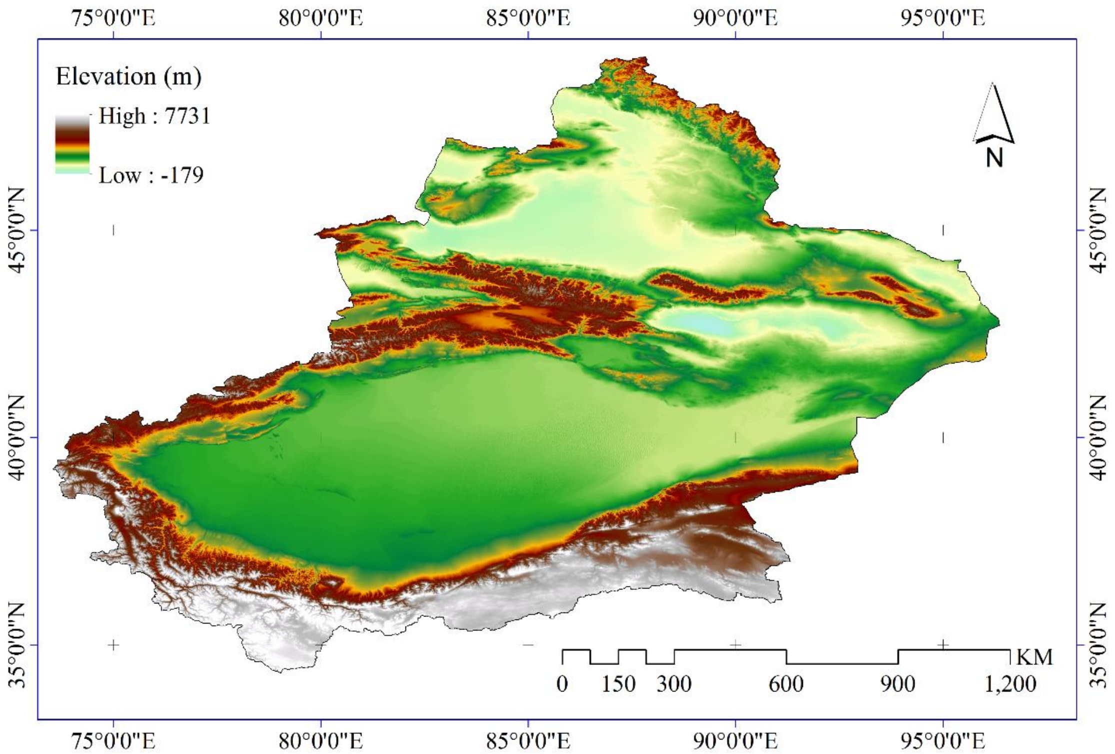

Fig. S6 Elevation. The map was plotted using ArcGIS 10.2 (http://www.esri.com/).

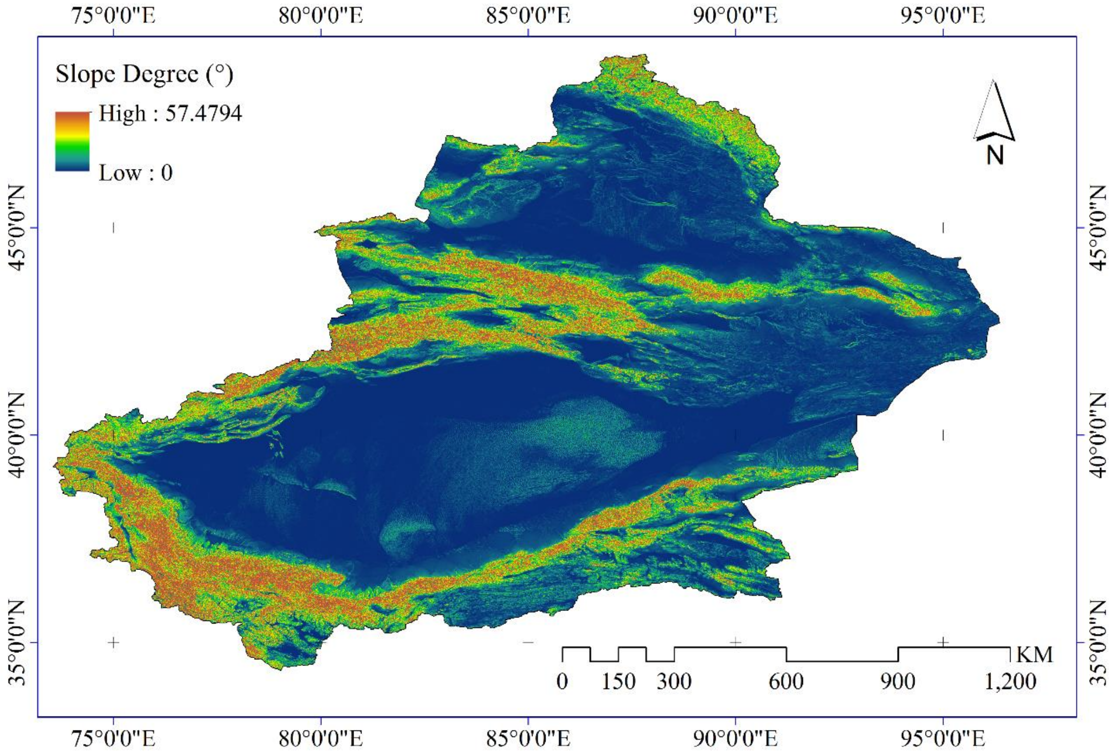

Fig. S7 Slope degree. The map was plotted using ArcGIS 10.2 (http://www.esri.com/).

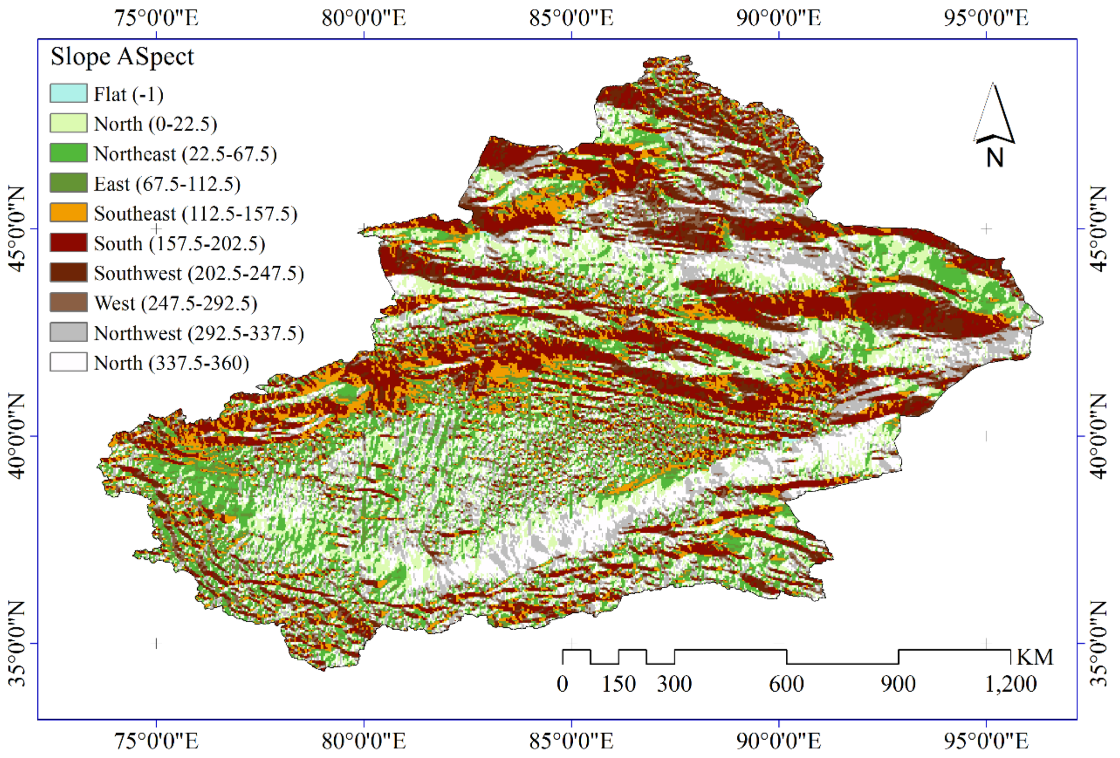

Fig. S8 Slop aspect. The map was plotted using ArcGIS 10.2 (http://www.esri.com/).

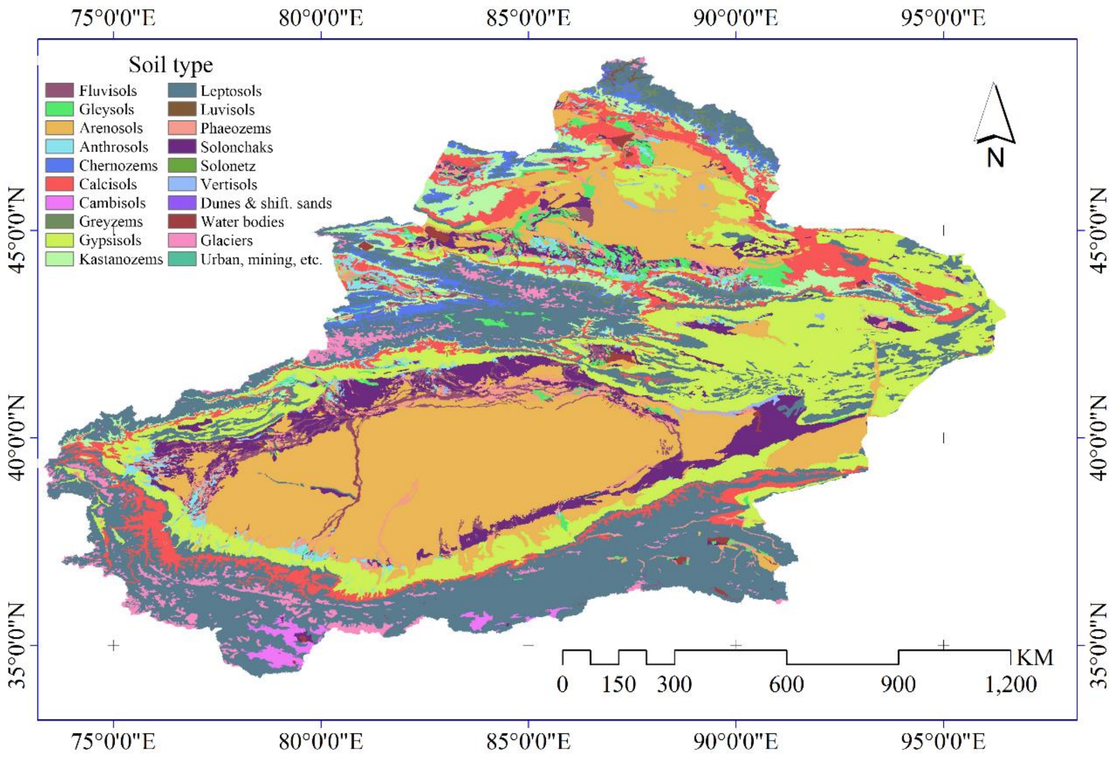

Fig. S9 Soil type. The map was plotted using ArcGIS 10.2 (http://www.esri.com/). Major soil groupings used for the HWSD map (Soil units: FAO90).

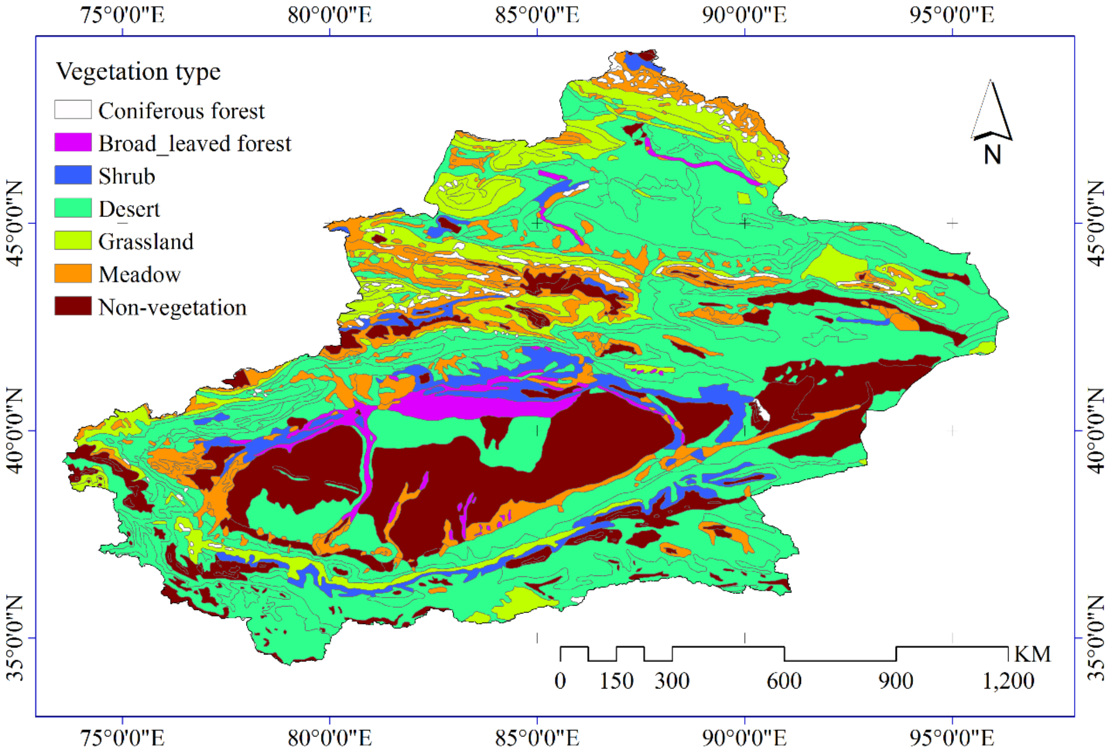

Fig. S10 Vegetation type. The map was plotted using ArcGIS 10.2 (http://www.esri.com/).
